# Hyperbaric Oxygen Therapy Achieved Fistula Healing in a Young Patient With Severe Refractory Perianal Crohn’s Disease

**DOI:** 10.7759/cureus.62987

**Published:** 2024-06-23

**Authors:** Justin Wen Hao Leong, Zhi Hao Yan, Fung Joon Foo, Frederick Hong-Xiang Koh, Lionel Tim-Ee Cheng, San Choon Kong, Tze Tong Tey

**Affiliations:** 1 Department of Gastroenterology and Hepatology, Sengkang General Hospital, Singapore, SGP; 2 Department of Colorectal Surgery, Sengkang General Hospital, Singapore, SGP; 3 Department of Diagnostic Radiology, Singapore General Hospital, Singapore, SGP

**Keywords:** hyperbaric medicine, inflammatory bowel disease, perianal fistula, hyperbaric oxygen treatment, crohn’s disease

## Abstract

The presence of perianal fistulae constitutes a more severe phenotype of Crohn’s disease (CD) that often requires intensive medical therapy, wound care, and surgical intervention. Despite therapeutic advances in inflammatory bowel disease, the treatment of perianal fistulae remains challenging. Hyperbaric oxygen therapy (HBOT) has been proposed as an adjunctive treatment modality for induction of fistula healing. We illustrate a case in which HBOT achieved fistula healing in a young patient with severe refractory perianal Crohn’s disease (pCD). We also review the current literature and discuss the role of HBOT in the treatment armamentarium of pCD.

## Introduction

Crohn’s disease (CD) is a chronic disease characterized by recurrent inflammation of the gastrointestinal tract. Perianal fistulae occur in 20-25% of patients with CD and constitute a more severe phenotype that often requires a combination of intensive medical therapy, wound care, and surgical intervention [1,2].

Hyperbaric oxygen therapy (HBOT) is a treatment modality that involves delivering 100% oxygen in a pressurized chamber at two to three times standard atmospheric pressure at sea level, where 1 atmosphere absolute (ATA) is defined as the average atmospheric pressure exerted at sea level. It represents an adjunctive treatment option in perianal Crohn’s disease (pCD) and is postulated to have beneficial effects on wound healing via stem cell mobilization, decrease in inflammatory cytokines, and reduction of wound hypoxia [3,4]. We report a case in which fistula healing was achieved with adjunctive HBOT.

## Case presentation

Our patient was a 21-year-old male of South Asian ethnicity with a background of ileocolonic CD with complex perianal fistulae. He was diagnosed with CD at 14 years old and first developed perianal fistulae at the age of 19 years. Since then, he has had multiple hospitalizations for recurrent perianal abscesses requiring both percutaneous and surgical drainage with seton insertion. He underwent two lines of biological treatment, which did not achieve fistula healing. First, he received infliximab but developed antibodies to infliximab after one year, with a secondary loss of response. Next, he received adalimumab but showed no improvement in inflammation and fistula discharge, suggesting primary non-response.

**Figure 1:**
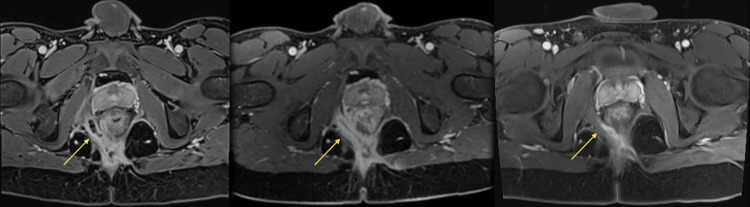
Serial axial T1-weighted contrast-enhanced MRI of the perianal region showing interval improvement of a curvilinear fluid-filled track (arrow) in the right ischioanal fossa over time. This track was one component of a complex perianal fistula with multiple openings. A (left): Appearance at first presentation. B (middle): Two months post treatment. C (right): Five months post treatment.

At this time, he was clinically malnourished with a body mass index (BMI) of 16.8. Laboratory results showed a hemoglobin (Hb) level of 9.5 g/dL, serum albumin level of 30 g/L, and C-reactive protein (CRP) level of 113.3 mg/L (normal: <3 mg/L). The perianal disease activity index (PDAI) score was 12. Magnetic resonance imaging of the pelvis showed a complex intersphincteric fistula with multiple internal openings (Figure 1).

In view of the refractory and debilitating disease with prior failure of two biologics, various options, including switching biologic therapy to ustekinumab (an interleukin-12/23 inhibitor), surgical treatment such as a diverting colostomy, and adjunctive treatment with HBOT, were considered. After discussion, a shared decision was made for ustekinumab with HBOT. He was started on ustekinumab with adjunctive HBOT concurrently (60 sessions, two-hour duration, 2.0 ATA) over a period of three months. He tolerated the HBOT well with no adverse events.

On review at 16 weeks after HBOT, clinical examination revealed complete fistula healing with the resolution of perineum swelling, closure of the external orifice, the absence of active perianal discharge, and radiological improvement (Figure 1). His PDAI score improved from 12 to 3 and his BMI increased from 16.8 to 19.4, with an improvement in his nutritional status. Biochemical response was demonstrated with CRP decreasing from 113.3 mg/L to 28.9 mg/L. Hb increased from 9.5 g/dL to 12.7 g/dL and albumin increased from 30 g/L to 38 g/L (Table 1). He reported an improvement in his quality of life in terms of his bowel movements, emotional well-being, and social functioning.

**Table 1:** Indices pre- and post-HBOT. HBOT: hyperbaric oxygen therapy.

	Prior to HBOT	16 weeks post HBOT
Hemoglobin (Hb)	9.5 g/dL	12.7 g/dL
Albumin	30 g/L	38 g/L
C-reactive protein (CRP)	113.3 mg/L	28.9 mg/L
Perianal disease activity index (PDAI)	12	3
Body mass index (BMI)	16.8	19.4

However, at nine months post-completion of HBOT, he presented with a recurrence of abdominal pain, diarrhea, and active perianal discharge. Repeat imaging showed active intestinal inflammation and recrudescence of a complex perianal fistula with abscess formation. This occurred despite compliance with maintenance therapy with ustekinumab. He was started on antibiotics and subsequently underwent percutaneous drainage of the abscess. While HBOT successfully induced fistula healing initially, the clinical response in this patient was not sustained.

## Discussion

Our case highlights a patient with severe refractory fistulizing pCD who achieved clinical remission and radiological improvement of his complex perianal fistulae with combined ustekinumab and adjunctive HBOT. Unfortunately, remission was not sustained after the conclusion of HBOT despite maintenance biologic therapy, suggesting that HBOT, rather than ustekinumab, was the major contributor to his initial therapeutic response.

Despite the multiple advances in the treatment of inflammatory bowel disease over the last decade, the treatment of perianal fistulae remains one of the biggest unmet needs in the treatment of CD. The implications of a diagnosis of pCD are myriad, including increased healthcare utilization, increased need for surgery, greater medication burden, and poorer quality of life [5].

Achieving closure of perianal fistulas is challenging. A recent study showed that this outcome was achieved in only 68% of patients at 18 months who underwent a combination of anti-tumor necrosis factor therapy and surgery [6-9]. HBOT presents the option of a novel adjunctive therapy with minimal adverse effects. Through breathing 100% oxygen under pressure, HBOT increases plasma and tissue oxygen levels, thereby relieving hypoxia and increasing the oxygen content of blood reaching inflamed bowel or chronic nonhealing fistulas and triggering tissue restorative pathways essential for wound healing. Additionally, HBOT has been shown to suppress the production of proinflammatory cytokines (IL-1, IL-6, and tumor necrosis factor-alpha) and modulate inflammatory responses by decreasing the ratio of CD4:CD8 T-cell subsets, reducing neutrophil chemotaxis and enhancing lymphocyte apoptosis. Lastly, relevant to pCD, antimicrobial pathways are also stimulated with the formation of reactive oxygen species. These pathways work in concert and contribute to the healing of the perianal fistula [10-12].

The emerging literature on the efficacy of HBOT in pCD is promising. Lansdorp et al. demonstrated a reduction in median PDAI score from 7.5 to 4 with an overall clinical response of 60% at 16 weeks after 40 daily sessions of HBOT in 20 patients with pCD who had failed conventional treatment [7]. Longer-term evaluation of the same group who received no further HBOT showed an increase in the biochemical indices of CRP and fecal calprotectin from week 16 to week 60, although PDAI scores and clinical closure response at 60 weeks were maintained. Further to this, a subsequent meta-analysis by McCurdy et al. showed that in patients with refractory pCD, HBOT was associated with an overall clinical response rate of 75% and a clinical remission rate of 55% [13].

Efficacy aside, safety remains an important determinant of a patient’s willingness to accept treatment. A recent review of patients undergoing HBOT showed an adverse effect rate of less than 1%. Middle ear or paranasal barotrauma was the most commonly reported adverse effect and was typically mild and self-resolving. Other adverse effects such as hyperbaric myopia, hypoglycemia, and claustrophobia have been reported but are rare. The majority of these events were minor and did not impact the continuation of treatment [14].

## Conclusions

The effectiveness of HBOT is heartening, particularly for the induction of healing of fistulizing disease. Achievement of clinical remission remains challenging and HBOT offers an adjunctive treatment option that is safe, well-tolerated, and effective. At present, there remains no consensus on the duration of treatment, and the optimal dose of HBOT remains uncertain. Further research is required to determine the optimal treatment duration, feasibility, cost-effectiveness, and long-term outcomes of HBOT and establish its place in the treatment armamentarium of pCD.
